# 3,3′-Diethyl-1,1′-(1,4-phenyl­ene­dimethyl­ene)diimidazol-3-ium bis­(hexa­fluoro­phosphate)

**DOI:** 10.1107/S1600536812028966

**Published:** 2012-06-30

**Authors:** Rosenani A. Haque, S. Fatimah Nasri, Mohd Mustaqim Rosli, Hoong-Kun Fun

**Affiliations:** a School of Chemical Sciences, Universiti Sains Malaysia, 11800 USM, Penang, Malaysia; b X-ray Crystallography Unit, School of Physics, Universiti Sains Malaysia, 11800 USM, Penang, Malaysia

## Abstract

In the title mol­ecular salt, C_18_H_24_N_4_^2+^·2PF_6_^−^, the complete dication is generated by a crystallographic inversion centre. The central benzene ring makes a dihedral angle of 77.19 (9)° with each of the imidazole rings. In the crystal, C—H⋯F inter­actions link the cations and anions into layers lying parallel to the *bc* plane. The hexa­fluoro­phosphate anion is disordered over two sets of sites in a 0.520 (11):0.480 (11) ratio.

## Related literature

For the properties of imidzole derivates, see: Shargel *et al.* (2006 ▶). For related structures, see: Haque *et al.* (2010 ▶, 2011 ▶).
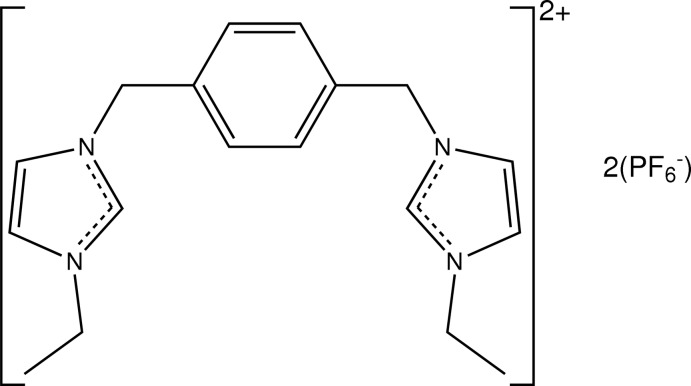


## Experimental

### 

#### Crystal data

C_18_H_24_N_4_^2+^·2PF_6_^−^
*M* *_r_* = 586.35
Triclinic, 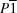

*a* = 8.5441 (5) Å
*b* = 8.6018 (5) Å
*c* = 9.5626 (6) Å
α = 67.913 (1)°
β = 77.928 (1)°
γ = 67.837 (1)°
*V* = 601.25 (6) Å^3^
*Z* = 1
Mo *K*α radiation
μ = 0.29 mm^−1^
*T* = 297 K
0.28 × 0.25 × 0.12 mm

#### Data collection

Bruker APEX DUO CCD diffractometer
Absorption correction: multi-scan (*SADABS*; Bruker, 2009 ▶) *T*_min_ = 0.925, *T*_max_ = 0.966
12274 measured reflections
3996 independent reflections
2968 reflections with *I* > 2σ(*I*)
*R*_int_ = 0.018

#### Refinement

*R*[*F* ^2^ > 2σ(*F* ^2^)] = 0.053
*wR*(*F* ^2^) = 0.170
*S* = 1.04
3996 reflections
228 parameters
21 restraints
H-atom parameters constrained
Δρ_max_ = 0.38 e Å^−3^
Δρ_min_ = −0.36 e Å^−3^

### 

Data collection: *APEX2* (Bruker, 2009 ▶); cell refinement: *SAINT* (Bruker, 2009 ▶); data reduction: *SAINT*; program(s) used to solve structure: *SHELXTL* (Sheldrick, 2008 ▶); program(s) used to refine structure: *SHELXTL*; molecular graphics: *SHELXTL*; software used to prepare material for publication: *SHELXTL* and *PLATON* (Spek, 2009 ▶).

## Supplementary Material

Crystal structure: contains datablock(s) I, global. DOI: 10.1107/S1600536812028966/hb6877sup1.cif

Structure factors: contains datablock(s) I. DOI: 10.1107/S1600536812028966/hb6877Isup2.hkl

Supplementary material file. DOI: 10.1107/S1600536812028966/hb6877Isup3.cml

Additional supplementary materials:  crystallographic information; 3D view; checkCIF report

## Figures and Tables

**Table 1:** Hydrogen-bond geometry (Å, °)

*D*—H⋯*A*	*D*—H	H⋯*A*	*D*⋯*A*	*D*—H⋯*A*
C3—H3*A*⋯F1^i^	0.93	2.49	3.408 (10)	170
C4—H4*A*⋯F3^ii^	0.93	2.48	3.369 (11)	160
C5—H5*A*⋯F6^iii^	0.93	2.32	3.211 (8)	159

Symmetry codes: (i) 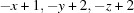; (ii) 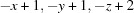; (iii) 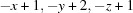.

**Fig. 1.**
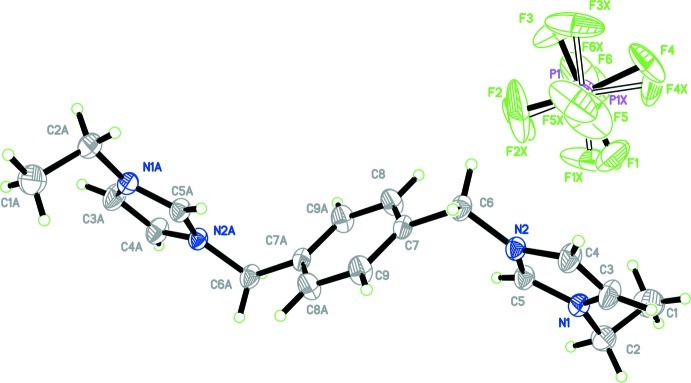
The molecular structure, showing 30% probability displacement ellipsoids. Hydrogen atoms are shown as spheres of arbitrary radius.

**Fig. 2.**
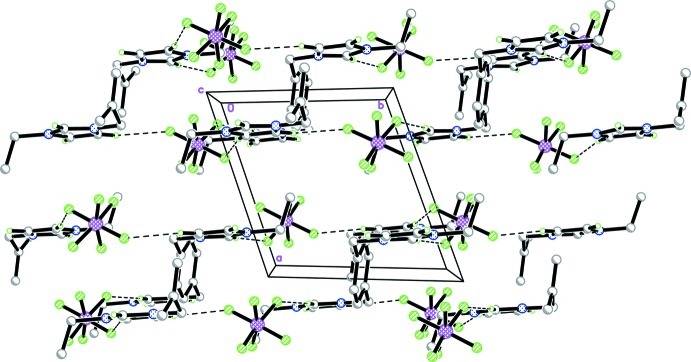
The crystal packing of (I). Dashed lines indicate hydrogen bonds. H atoms not involved in the hydrogen bond interactions have been omitted for clarity.

## supplementary crystallographic information

### Comment

Substituted imidazole derivatives are valuable in the treatment of many systemic fungal infections (Shargel *et al.*, 2006). Previously, we have reported crystal structures of *para*- xylyl linked bis-imidazolium salts with propyl (Haque *et al.*, 2011) and benzyl (Haque *et al.*, 2010) substitutions. In this report, we describe the crystal structure of a *para*-xylyl linked bis-benzimidazolium salt with ethyl substitutuents

All parameters in (I) are within normal ranges. The complete dication is generated by a crystallographic inversion centre. The central benzene ring (C7—C9/C7A—C9A) makes a dihedral angle of 77.19 (9)° with the imidazole rings (N1—N2/C3—C5 and N1A—N2A/C3A—C5A). The hexafluorophosphate anions are disordered over two sets of sites with the final refined occupancies of 0.52 (1):0.48 (1).

In the crystal, C3—H3A···F1^i^, C4—H4A···F3^ii^ and C5—H5A···F6^iii^ (Table 1) interactions link the molecules into layers lying parallel to the *bc*-plane.

### Experimental

To a solution of 1,4-bis((1*H*-imidazol-1-yl)methyl)benzene (1.00 g, 0.004 mol) in 15 ml of acetonitrile, 1-iodoethane (1.31 g, 0.008 mol) was added. The mixture was refluxed at 363 K for 24 h. The resultant white precipitate was filtered, washed with fresh acetonitrile (2 × 5 ml) and converted directly to its hexafluorophosphate counterpart by metathesis reaction using KPF_6_ (1.67 g, 0.008 mol) in 40 ml of methanol/water. The white precipitates were collected, washed with fresh acetonitrile (2 × 3 ml) to give the product as a white solid (1.78 g, 67%). *M*.p 467–469 K. Colourless blocks were obtained by slow diffusion method of the salt solution by using diethyl ether and acetonitrile at room temperature.

### Refinement

All H atoms attached to C atoms were fixed geometrically and refined as riding with C—H = 0.93–0.97 Å and *U*_iso_(H) = 1.2*U*_eq_(C) or 1.5*U*_eq_(C) for methyl H atoms. A rotating group model was applied to the methyl group. The hexafluorophosphate anion is disordered over two sets of sites with refined occupancies of 0.52 (1):0.48 (1).

### Crystal data

**Table:**

C_18_H_24_N_4_^2+^·2PF_6_^−^	*Z* = 1
*M**_r_* = 586.35	*F*(000) = 298
Triclinic, *P*1	*D*_x_ = 1.619 Mg m^−^^3^
Hall symbol: -P 1	Mo *K*α radiation, λ = 0.71073 Å
*a* = 8.5441 (5) Å	Cell parameters from 3921 reflections
*b* = 8.6018 (5) Å	θ = 2.3–29.3°
*c* = 9.5626 (6) Å	µ = 0.29 mm^−^^1^
α = 67.913 (1)°	*T* = 297 K
β = 77.928 (1)°	Block, colourless
γ = 67.837 (1)°	0.28 × 0.25 × 0.12 mm
*V* = 601.25 (6) Å^3^	

### Data collection

**Table:**

Bruker APEX DUO CCD diffractometer	3996 independent reflections
Radiation source: fine-focus sealed tube	2968 reflections with *I* > 2σ(*I*)
Graphite monochromator	*R*_int_ = 0.018
φ and ω scans	θ_max_ = 31.7°, θ_min_ = 2.3°
Absorption correction: multi-scan (*SADABS*; Bruker, 2009)	*h* = −12→12
*T*_min_ = 0.925, *T*_max_ = 0.966	*k* = −12→12
12274 measured reflections	*l* = −14→13

### Refinement

**Table:**

Refinement on *F*^2^	Primary atom site location: structure-invariant direct methods
Least-squares matrix: full	Secondary atom site location: difference Fourier map
*R*[*F*^2^ > 2σ(*F*^2^)] = 0.053	Hydrogen site location: inferred from neighbouring sites
*wR*(*F*^2^) = 0.170	H-atom parameters constrained
*S* = 1.04	*w* = 1/[σ^2^(*F*_o_^2^) + (0.087*P*)^2^ + 0.1678*P*] where *P* = (*F*_o_^2^ + 2*F*_c_^2^)/3
3996 reflections	(Δ/σ)_max_ = 0.026
228 parameters	Δρ_max_ = 0.38 e Å^−^^3^
21 restraints	Δρ_min_ = −0.36 e Å^−^^3^

### Special details

**Table:**

Geometry. All e.s.d.'s (except the e.s.d. in the dihedral angle between two l.s. planes) are estimated using the full covariance matrix. The cell e.s.d.'s are taken into account individually in the estimation of e.s.d.'s in distances, angles and torsion angles; correlations between e.s.d.'s in cell parameters are only used when they are defined by crystal symmetry. An approximate (isotropic) treatment of cell e.s.d.'s is used for estimating e.s.d.'s involving l.s. planes.
Refinement. Refinement of *F*^2^ against ALL reflections. The weighted *R*-factor *wR* and goodness of fit *S* are based on *F*^2^, conventional *R*-factors *R* are based on *F*, with *F* set to zero for negative *F*^2^. The threshold expression of *F*^2^ > σ(*F*^2^) is used only for calculating *R*-factors(gt) *etc*. and is not relevant to the choice of reflections for refinement. *R*-factors based on *F*^2^ are statistically about twice as large as those based on *F*, and *R*- factors based on ALL data will be even larger.

### Fractional atomic coordinates and isotropic or equivalent isotropic displacement parameters (Å^2^)

**Table:**

	*x*	*y*	*z*	*U*_iso_*/*U*_eq_	Occ. (<1)
P1	0.2804 (5)	0.8326 (6)	0.7615 (5)	0.0638 (15)	0.520 (11)
F1	0.3579 (13)	0.9624 (13)	0.7737 (9)	0.131 (3)	0.520 (11)
F2	0.4136 (13)	0.7509 (17)	0.6579 (12)	0.199 (7)	0.520 (11)
F3	0.2044 (14)	0.6984 (10)	0.7644 (13)	0.163 (4)	0.520 (11)
F4	0.1422 (6)	0.8696 (10)	0.8921 (5)	0.107 (3)	0.520 (11)
F5	0.3982 (12)	0.7149 (17)	0.8932 (9)	0.166 (5)	0.520 (11)
F6	0.1612 (11)	0.9775 (11)	0.6426 (9)	0.114 (3)	0.520 (11)
P1X	0.2841 (4)	0.8298 (4)	0.7654 (4)	0.0360 (7)	0.480 (11)
F1X	0.3854 (13)	0.9578 (14)	0.718 (2)	0.203 (6)	0.480 (11)
F2X	0.4179 (9)	0.7801 (15)	0.6381 (8)	0.125 (4)	0.480 (11)
F3X	0.1751 (10)	0.7065 (9)	0.8061 (13)	0.142 (5)	0.480 (11)
F4X	0.1575 (11)	0.9329 (18)	0.8653 (13)	0.176 (5)	0.480 (11)
F5X	0.3981 (10)	0.6706 (12)	0.8876 (10)	0.124 (3)	0.480 (11)
F6X	0.1650 (13)	0.9487 (15)	0.6319 (9)	0.123 (4)	0.480 (11)
N1	0.7726 (2)	0.9473 (2)	0.78904 (17)	0.0434 (3)	
N2	0.80090 (18)	0.67771 (18)	0.81853 (15)	0.0376 (3)	
C1	0.5791 (3)	1.2530 (3)	0.7237 (4)	0.0789 (8)	
H1A	0.5718	1.3735	0.6648	0.118*	
H1B	0.5405	1.2453	0.8271	0.118*	
H1C	0.5094	1.2165	0.6841	0.118*	
C2	0.7574 (3)	1.1356 (3)	0.7159 (3)	0.0580 (5)	
H2A	0.8010	1.1559	0.6108	0.070*	
H2B	0.8255	1.1650	0.7653	0.070*	
C3	0.7587 (3)	0.8606 (3)	0.9407 (2)	0.0568 (5)	
H3A	0.7410	0.9089	1.0171	0.068*	
C4	0.7751 (3)	0.6920 (3)	0.9598 (2)	0.0512 (5)	
H4A	0.7700	0.6025	1.0515	0.061*	
C5	0.7975 (2)	0.8345 (2)	0.71706 (19)	0.0424 (4)	
H5A	0.8106	0.8607	0.6126	0.051*	
C6	0.8154 (3)	0.5168 (2)	0.78841 (19)	0.0460 (4)	
H6A	0.7027	0.5143	0.7890	0.055*	
H6B	0.8726	0.4127	0.8689	0.055*	
C7	0.9118 (2)	0.5083 (2)	0.63878 (17)	0.0375 (3)	
C8	0.8268 (2)	0.5812 (3)	0.5089 (2)	0.0513 (5)	
H8A	0.7099	0.6362	0.5139	0.062*	
C9	1.0854 (3)	0.4272 (3)	0.6285 (2)	0.0512 (5)	
H9A	1.1440	0.3775	0.7151	0.061*	

### Atomic displacement parameters (Å^2^)

**Table:**

	*U*^11^	*U*^22^	*U*^33^	*U*^12^	*U*^13^	*U*^23^
P1	0.061 (2)	0.066 (2)	0.052 (2)	−0.0193 (18)	−0.0073 (16)	−0.0066 (16)
F1	0.183 (8)	0.157 (6)	0.133 (5)	−0.118 (5)	0.029 (4)	−0.093 (5)
F2	0.131 (7)	0.242 (11)	0.180 (9)	0.076 (7)	−0.016 (6)	−0.158 (8)
F3	0.213 (9)	0.120 (5)	0.224 (8)	−0.102 (5)	−0.040 (7)	−0.070 (6)
F4	0.060 (2)	0.168 (5)	0.049 (2)	−0.027 (3)	0.0210 (15)	−0.013 (3)
F5	0.090 (5)	0.284 (12)	0.084 (4)	−0.030 (6)	−0.052 (4)	−0.026 (6)
F6	0.084 (4)	0.078 (3)	0.087 (5)	0.005 (2)	0.005 (3)	0.035 (3)
P1X	0.0345 (13)	0.0375 (13)	0.0373 (15)	−0.0150 (10)	0.0042 (10)	−0.0143 (11)
F1X	0.102 (5)	0.148 (7)	0.343 (16)	−0.096 (5)	−0.007 (7)	−0.013 (8)
F2X	0.073 (4)	0.213 (9)	0.061 (3)	−0.023 (5)	0.027 (3)	−0.055 (4)
F3X	0.088 (3)	0.092 (4)	0.196 (8)	−0.060 (3)	−0.062 (4)	0.064 (5)
F4X	0.115 (5)	0.273 (10)	0.239 (9)	−0.035 (6)	0.011 (5)	−0.231 (9)
F5X	0.070 (4)	0.105 (4)	0.107 (5)	−0.005 (3)	−0.011 (3)	0.041 (3)
F6X	0.089 (5)	0.178 (9)	0.048 (3)	−0.007 (5)	−0.026 (3)	−0.004 (4)
N1	0.0464 (8)	0.0408 (7)	0.0481 (8)	−0.0170 (6)	0.0016 (6)	−0.0202 (6)
N2	0.0450 (7)	0.0383 (7)	0.0311 (6)	−0.0158 (5)	0.0033 (5)	−0.0145 (5)
C1	0.0633 (15)	0.0511 (12)	0.114 (2)	−0.0112 (11)	−0.0112 (14)	−0.0229 (14)
C2	0.0608 (12)	0.0401 (9)	0.0745 (14)	−0.0204 (8)	0.0033 (10)	−0.0211 (9)
C3	0.0789 (14)	0.0595 (12)	0.0443 (10)	−0.0269 (10)	0.0003 (9)	−0.0286 (9)
C4	0.0739 (13)	0.0520 (10)	0.0319 (8)	−0.0240 (9)	−0.0003 (8)	−0.0170 (7)
C5	0.0519 (9)	0.0406 (8)	0.0356 (7)	−0.0179 (7)	0.0035 (6)	−0.0146 (6)
C6	0.0636 (11)	0.0412 (8)	0.0374 (8)	−0.0244 (8)	0.0100 (7)	−0.0177 (7)
C7	0.0481 (9)	0.0326 (7)	0.0328 (7)	−0.0135 (6)	0.0019 (6)	−0.0143 (6)
C8	0.0389 (9)	0.0648 (12)	0.0444 (9)	−0.0058 (8)	−0.0033 (7)	−0.0231 (9)
C9	0.0506 (10)	0.0611 (11)	0.0360 (8)	−0.0064 (8)	−0.0097 (7)	−0.0181 (8)

### Geometric parameters (Å, º)

**Table:**

P1—F2	1.490 (7)	C1—H1A	0.9600
P1—F3	1.513 (6)	C1—H1B	0.9600
P1—F6	1.531 (7)	C1—H1C	0.9600
P1—F1	1.545 (6)	C2—H2A	0.9700
P1—F5	1.558 (7)	C2—H2B	0.9700
P1—F4	1.566 (5)	C3—C4	1.347 (3)
P1X—F4X	1.533 (6)	C3—H3A	0.9300
P1X—F1X	1.532 (7)	C4—H4A	0.9300
P1X—F2X	1.553 (6)	C5—H5A	0.9300
P1X—F3X	1.557 (6)	C6—C7	1.507 (2)
P1X—F5X	1.567 (6)	C6—H6A	0.9700
P1X—F6X	1.577 (6)	C6—H6B	0.9700
N1—C5	1.321 (2)	C7—C9	1.381 (3)
N1—C3	1.362 (3)	C7—C8	1.382 (2)
N1—C2	1.469 (2)	C8—C9^i^	1.383 (2)
N2—C5	1.327 (2)	C8—H8A	0.9300
N2—C4	1.367 (2)	C9—C8^i^	1.383 (2)
N2—C6	1.473 (2)	C9—H9A	0.9300
C1—C2	1.482 (3)		
			
F2—P1—F3	82.7 (7)	C2—C1—H1A	109.5
F2—P1—F6	98.2 (6)	C2—C1—H1B	109.5
F3—P1—F6	90.4 (5)	H1A—C1—H1B	109.5
F2—P1—F1	100.0 (7)	C2—C1—H1C	109.5
F3—P1—F1	175.0 (6)	H1A—C1—H1C	109.5
F6—P1—F1	93.3 (6)	H1B—C1—H1C	109.5
F2—P1—F5	88.9 (6)	N1—C2—C1	111.63 (19)
F3—P1—F5	98.9 (7)	N1—C2—H2A	109.3
F6—P1—F5	169.0 (8)	C1—C2—H2A	109.3
F1—P1—F5	77.1 (6)	N1—C2—H2B	109.3
F2—P1—F4	165.7 (7)	C1—C2—H2B	109.3
F3—P1—F4	86.3 (5)	H2A—C2—H2B	108.0
F6—P1—F4	90.8 (5)	C4—C3—N1	107.53 (16)
F1—P1—F4	90.4 (4)	C4—C3—H3A	126.2
F5—P1—F4	83.8 (5)	N1—C3—H3A	126.2
F4X—P1X—F1X	90.9 (6)	C3—C4—N2	106.79 (17)
F4X—P1X—F2X	163.0 (8)	C3—C4—H4A	126.6
F1X—P1X—F2X	74.1 (7)	N2—C4—H4A	126.6
F4X—P1X—F3X	89.2 (5)	N1—C5—N2	108.71 (15)
F1X—P1X—F3X	176.8 (6)	N1—C5—H5A	125.6
F2X—P1X—F3X	105.3 (6)	N2—C5—H5A	125.6
F4X—P1X—F5X	100.5 (7)	N2—C6—C7	112.44 (14)
F1X—P1X—F5X	99.7 (6)	N2—C6—H6A	109.1
F2X—P1X—F5X	90.0 (5)	C7—C6—H6A	109.1
F3X—P1X—F5X	83.5 (5)	N2—C6—H6B	109.1
F4X—P1X—F6X	88.6 (6)	C7—C6—H6B	109.1
F1X—P1X—F6X	92.3 (7)	H6A—C6—H6B	107.8
F2X—P1X—F6X	84.2 (6)	C9—C7—C8	118.74 (15)
F3X—P1X—F6X	84.5 (5)	C9—C7—C6	121.03 (16)
F5X—P1X—F6X	164.7 (7)	C8—C7—C6	120.23 (17)
C5—N1—C3	108.48 (15)	C9^i^—C8—C7	120.44 (17)
C5—N1—C2	125.15 (17)	C9^i^—C8—H8A	119.8
C3—N1—C2	126.35 (17)	C7—C8—H8A	119.8
C5—N2—C4	108.49 (15)	C7—C9—C8^i^	120.82 (16)
C5—N2—C6	127.06 (14)	C7—C9—H9A	119.6
C4—N2—C6	124.27 (15)	C8^i^—C9—H9A	119.6
			
C5—N1—C2—C1	104.3 (3)	C6—N2—C5—N1	176.08 (17)
C3—N1—C2—C1	−73.6 (3)	C5—N2—C6—C7	29.1 (3)
C5—N1—C3—C4	−0.2 (3)	C4—N2—C6—C7	−156.30 (18)
C2—N1—C3—C4	178.0 (2)	N2—C6—C7—C9	90.2 (2)
N1—C3—C4—N2	0.6 (3)	N2—C6—C7—C8	−90.3 (2)
C5—N2—C4—C3	−0.9 (2)	C9—C7—C8—C9^i^	−0.1 (3)
C6—N2—C4—C3	−176.35 (19)	C6—C7—C8—C9^i^	−179.61 (18)
C3—N1—C5—N2	−0.4 (2)	C8—C7—C9—C8^i^	0.1 (3)
C2—N1—C5—N2	−178.59 (18)	C6—C7—C9—C8^i^	179.61 (18)
C4—N2—C5—N1	0.8 (2)		

Symmetry code: (i) −*x*+2, −*y*+1, −*z*+1.

### Hydrogen-bond geometry (Å, º)

**Table:**

*D*—H···*A*	*D*—H	H···*A*	*D*···*A*	*D*—H···*A*
C3—H3*A*···F1^ii^	0.93	2.49	3.408 (10)	170
C4—H4*A*···F3^iii^	0.93	2.48	3.369 (11)	160
C5—H5*A*···F6^iv^	0.93	2.32	3.211 (8)	159

Symmetry codes: (ii) −*x*+1, −*y*+2, −*z*+2; (iii) −*x*+1, −*y*+1, −*z*+2; (iv) −*x*+1, −*y*+2, −*z*+1.
